# Usefulness of Blood Biomarkers in Screening Patients with Obstructive Sleep Apnea: Could Albumin Indices and Uric Acid-to-HDL Ratio Be New OSAS Severity Indices?

**DOI:** 10.3390/arm93050042

**Published:** 2025-10-07

**Authors:** Mihrican Yeşildağ, Taha Tahir Bekçi

**Affiliations:** Department of Chest Diseases, S.B University Konya Training and Research Hospital, 42090 Konya, Turkey; tahabekci@yahoo.com

**Keywords:** obstructive sleep apnea syndrome, screening, hematological indices, uric acid–HDL ratio, RDW–albumin ratio

## Abstract

**Highlights:**

**What are the main findings?**

- Hematological parameters showed significant differences in the presence and severity of OSAS.
- BAR, RAR, and UHR emerged as important hematological indices associated with OSAS.

**What is the implication of the main finding?**

- Hematological parameters can be used as simple and low-cost biomarkers for the early prediction of OSAS.
- In clinical settings with limited access to PSG, hematological indices can guide the prioritization and decision-making process for patients who require referral for PSG.

**Abstract:**

Background and Objectives: Hematological parameters are increasingly being investigated as readily accessible biomarkers for the diagnosis of obstructive sleep apnea syndrome (OSAS). In our study, we aimed to investigate the relationship between OSAS and albumin indices and the uric acid-to-HDL ratio (UHR). Methods: The demographic and laboratory data and AHI (apnea–hypopnea index) values of 613 patients who underwent polysomnography were obtained retrospectively from their files. Blood parameters such as white blood cells (WBCs), red blood cell distribution width (RDW), red blood cells (RBCs), hemoglobin (Hb), hematocrit (Hct), platelets (PLTs), C-reactive protein (CRP), albumin, blood urea nitrogen (BUN), and high-density lipoproteins (HDLs) were obtained from the files. Laboratory indices such as the BUN-to-albumin ratio (BAR), neutrophil-to-albumin ratio (NAR), RDW-to-albumin ratio (RAR), CRP-to-albumin ratio (CAR), and UHR were calculated. OSAS was categorized as simple snoring (SS) (control) (AHI < 5), mild (5 ≤ AHI < 15), moderate (15 ≤ AHI < 30), and severe (AHI ≥ 30). The patients were also grouped as severe (AHI ≥ 30) and non-severe (5 > AHI < 30) OSAS and compared in terms of laboratory parameters and indices. Results: Of the 613 participants, 366 (59.7%) were men, and the average age of participants was 55.22 ± 11.13 years. The biomarkers such as RBCs, Hb, Htc, CRP, BUN, creatinine, uric acid, HDLs, CAR, RAR, BAR, and UHR showed significant differences between OSAS patients and controls. WBCs, basophils, RBCs, RDW, Htc, PLTs, HDLs, uric acid, RAR, NAR, and UHR indices were significantly different between the severe OSAS and non-severe OSAS groups (*p* < 0.05). BAR (OR = 1.151; CI = 1.056 − 1.256; *p* = 0.001) and UHR (OR = 2.257; 95% CI = 1.507 − 3.382; *p* < 0.001) were the most important indices predicting OSAS, while RAR (OR = 1.844; CI = 1.224 − 2.778; *p* = 0.003) and UHR (OR = 2.203; 95% CI = 1.496 − 3.243; *p* < 0.001) were the strongest indices associated with severe OSAS. Conclusion: In our study, RAR, BAR, and UHR indices were closely associated with the presence and severity of OSAS. These indices can be considered low-cost, readily available methods for predicting OSAS patients.

## 1. Introduction

Obstructive sleep apnea syndrome (OSAS) is increasing and becoming an important public health problem [1]. It causes increased morbidity and mortality associated with many systems and disorders [2]. However, the underlying mechanism of OSAS is still unknown. However, an increase in serum inflammatory markers has been reported, mainly in patients with severe OSAS [3]. The cause-and-effect relationship between intermittent hypoxia and chronic inflammatory processes, which are involved in the pathogenesis of OSAS, may be responsible for the mechanism [4]. Intermittent hypoxia causes systemic oxidative stress, metabolic changes, and activation of pro-inflammatory factors in OSAS [5], leading to systemic and local inflammation [6]. These mechanisms are also responsible for cardiovascular health problems and mortality in individuals with OSAS [7]. Recent research has proposed that various inflammatory biomarkers are also related to disease or disease severity [8,9].

Although OSAS is a very common disease, most patients with OSAS remain undiagnosed and untreated. When untreated, patients with OSAS have increased mortality, morbidity, and comorbidities [6,10]. Although PSG is the definitive screening method for OSAS, the fact that it requires a long time and specialized equipment has increased the search for new, cheaper, and more accessible biomarkers [11,12].

The American Academy of Sleep Medicine (AASM) clinical practice guidelines [6] report that simple, inexpensive, easily accessible, clinically sensitive, and specific screening tests, such as hematological parameters, are essential screening methods to better predict the diagnosis and severity of OSAS [13]. Numerous blood biomarkers have been investigated in OSAS [14]. Among these, hematological indices such as neutrophil count, lymphocyte count, red blood cell distribution width (RDW), mean platelet volume (MPV), white blood cells (WBCs), and platelet distribution width (PDW) have been recommended as alternative diagnostic markers of the disease, such as C-reactive protein (CRP), to assess inflammatory burden in OSAS [15]. The neutrophil-to-lymphocyte ratio (NLR) (which has been reported as a prognostic marker in many studies), CRP (an acute-phase protein), albumin (a negative acute-phase protein that is negatively associated with the severity of inflammatory responses), and the CRP-to-albumin ratio (CAR) (which is obtained by proportioning them) are still being investigated with great interest [16]. Some studies suggest that there is a relationship between OSAS and platelet activation and that there may be a relationship between platelet aggregation and OSAS [17]. The NLR is an inexpensive, readily available inflammatory biomarker whose role in various inflammatory conditions has been explored. However, although some studies have shown that the NLR is increased in severe OSAS [18], some studies have not found a correlation between the NLR and the presence or severity of OSAS [19].

Monocytes and macrophages also have an enormously important function in the inflammatory response and contribute to the release of pro-inflammatory cytokines at sites of inflamed tissue. High-density lipoprotein (HDL) cholesterol has an anti-inflammatory mechanism, and the monocyte-to-HDL cholesterol ratio (MHR) is a newly recognized biomarker of inflammation [9].

In addition to albumin, an important acute-phase reactant [20,21], albumin indices such as the CAR, the BUN-to-albumin ratio (BAR), the neutrophil-to-albumin ratio (NAR), the RDW-to-albumin ratio (RAR), uric acid and the uric acid-to-HDL ratio (UHR) (which are important oxidative stress markers), RDW (a periodical blood test related to chronic hypoxia), and increased hemoglobin (Hb) levels are thought to be associated with intermittent hypoxia in OSAS [22]. The Hb-to-RBC ratio (HRR) [23,24] is a recently studied index in chronic inflammatory events.

The lack of standardized and validated biomarkers for OSAS screening has led to an increase in research in this area. Many parameters, such as Hb, WBCs, hematocrit (Htc), mean corpuscular volume (MCV), PDW, MPV, RDW, and platelets, can be readily available from peripheral blood complete blood count (CBC) analysis. Furthermore, many parameters, such as albumin, uric acid, HDL, and LDL, can be easily obtained from blood biochemical analyses. In addition, many laboratory indices, such as the NLR and platelet-to-lymphocyte ratio (PLR), BAR, CAR, NAR, RAR, and UHR, which are obtained by proportioning many blood parameters to each other, can provide information about the disease. However, the diagnostic utility of these biomarkers and indices and their combinations in defining OSAS is uncertain [24].

This study aimed to evaluate the relationship between the AHI and laboratory parameters, as well as the indices obtained by proportioning them to each other in patients with suspected OSAS, and to investigate valuable blood biomarkers in the diagnosis, severity, and screening of OSAS.

In conclusion, we aimed to examine the biomarkers that may answer the question ‘who should be prioritized?’ in the referral of patients to PSG, which is the gold standard in OSAS but is expensive, difficult to access, and requires experienced equipment.

## 2. Materials and Methods

### 2.1. Study Protocol and Ethical Approval

This retrospective study was analyzed by examining the medical files of patients who were subjected to polysomnography (PSG) in the Sleep Unit of the Health Sciences University Konya Training and Research Hospital. The Institutional Ethics Committee of the University of Health Sciences Konya Training and Research Hospital’s Medical Specialty Education Board approved this study per the Declaration of Helsinki and Good Clinical Practice Guidelines, with its decision dated 3 January 2019 and numbered 01–12 (approval number: 48929119/774).

### 2.2. Study Participants

The medical files of 775 patients whose sleep-related data were collected in the PSG unit were retrospectively analyzed. The participants’ demographic data, laboratory data, and AHI values were retrospectively recorded from their files. After excluding patients under the age of 18, pregnant women, patients with sleep disorders other than OSAS, patients with poor sleep efficiency, patients who were unable to complete the PSG examination, and patients with incomplete laboratory blood values or no laboratory data, 613 patients who met our criteria were included in this study. Patients were categorized as simple snoring (SS) (control) (AHI < 5), mild (5≤ AHI < 15), moderate (15≤ AHI < 30), and severe (AHI ≥30) OSAS [25]. They were also categorized as OSAS with (AHI >5) and without (AHI < 5), and severe (AHI ≥30) and non-severe OSAS (AHI < 30). These patient groups were then analyzed for many laboratory parameters and indices for the diagnosis, severity, and screening of OSAS.

### 2.3. Polysomnography Studies

All participants underwent a single night of PSG. The recordings were performed using Alice 6 LDE Sleepware, a USA-brand PSG device, in a separate room, at night, accompanied by an attendant. Measurements were performed using four channels: electrooculography, electroencephalography, submental and tibial electromyography, and electrocardiogram, as well as PSG elements such as an oronasal airflow sensor, a body position sensor, abdominal and chest motion sensors, a tracheal microphone, and a pulse oximeter. The AHI was defined as the sum of apnea and hypopnea events recorded within one hour. It was defined according to the American Academy of Sleep Medicine (AASM) guidelines as apnea (a decrease in airflow of at least 90% of the baseline amplitude for ten seconds) and hypopnea (a decrease in airflow of at least 30% of the baseline amplitude for ten seconds with at least 3% oxygen desaturation or arousal) and the oxygen desaturation index (a 3% or greater fall in oxygen saturation during sleep). In the determination of the diagnosis and severity of OSAS, simple snoring (SS) (control) was categorized as AHI < 5, mild (5≤ AHI < 15), moderate (15≤ AHI < 30), and severe OSAS (AHI ≥30), according to the AHI values, considering the AASM guidelines [25].

### 2.4. Laboratory Studies

Whole-blood parameters and laboratory parameters were collected from hospital medical records and medical files after obtaining permission from the local Research Ethics Committee. The blood parameters studied included neutrophils, lymphocytes, monocytes, basophils, eosinophils, WBCs, RDW, RBCs, Hb, Hct, PDW, MCV, MPV, PLTs, CRP, albumin, uric acid, BUN, creatinine, HDLs, LDLs, NLR, PLR, RDW–albumin ratio (RAR), CRP–albumin ratio (CAR), BUN–albumin ratio (BAR), neutrophil–albumin ratio (NAR), monocyte–HDL ratio (MHR), monocyte–LDL ratio (MLR), uric acid–HDL ratio (UHR), and hemoglobin–RBC ratio (HRR). These values were compared with the AHI and laboratory parameters. Moreover, indices that can be used for the diagnosis, severity, and screening of OSAS were investigated.

### 2.5. Statistical Analysis

Statistical calculations were analyzed with the IBM SPSS Statistics for Windows–version 22.0 software. The descriptive statistical variables were presented as frequencies and percentages for the categorical data and as means ± standard deviations (SDs) or medians + IQRs for the continuous data. The normality of the values was analyzed with the Kolmogorov–Smirnov and Shapiro–Wilk tests. In the comparison of OSAS groups, parametric tests (independent *t*-tests) were applied to analyze continuous variables with a normal data distribution, and non-parametric tests (Mann–Whitney U and Kruskal–Wallis) were applied for variables without a normal data distribution. Variables that could potentially predict the presence and severity of OSAS were analyzed by creating a binary logistic regression model. A statistical value of less than 0.05 was evaluated as statistically valid.

## 3. Results

We analyzed 613 individuals who had undergone a polysomnogram with a pre-diagnosis of OSAS. In total, 431 patients were diagnosed with OSAS, while 182 patients were considered to have simple snoring (control). Mild, moderate, and severe OSAS consisted of 176, 116, and 139 patients, respectively. A total of 366 (59.7%) of the patients were male. Age and male gender were statistically highly significant in the OSAS patient group (*p* < 0.05). Statistically, OSAS patients consisted of older male patients. Demographic characteristics and laboratory analyses of the patients in the control and OSAS patient groups are presented in Table 1. The average age of the subjects diagnosed with OSAS was 55.22 ± 11.13 years, while it was 49.02 ± 12.02 years in the simple snoring group. There was a statistically significant difference between the groups (*p* < 0.001). The mean ages of mild OSAS (54.57 ± 11.02), moderate OSAS (54.49 ± 11.24), and severe OSAS (56.00 ± 11.11) patients increased with OSAS severity and were significantly different (*p* < 0.001). Comprehensive analyses of OSAS and non-OSAS, OSAS severity groups, and severe and non-severe OSAS groups in terms of laboratory parameters and indices are presented in Table 1, Table 2 and Table 3. Statistically significant findings are emphasized, and parameters that did not show a significant relationship with disease diagnosis and severity are also described.

When evaluating OSAS and non-OSAS patient groups, RBC, Hb, Htc, CRP, BUN, creatinine, uric acid, CAR, RAR, BAR, and UHR values were significantly higher in the OSAS group, while HDL was significantly lower (*p* < 0.05) (Table 1).

When the OSAS severity groups were compared, the WBC, RBC, RDW, Htc, CRP, BUN, creatinine, albumin, uric acid, RAR, BAR, and UHR parameters were significantly different (*p* < 0.05) (Table 2).

In the differentiation of severe OSAS, WBCs, neutrophils, basophils, RBCs, RDW, Htc, PLTs, HDLs, uric acid, RAR, NAR, and UHR were statistically significant biomarkers (*p* < 0.05) (Table 3).

Multivariate logistic regression analysis was performed to determine the predictors of OSAS diagnosis and severe OSAS, as shown in Table 4. Among the laboratory indices, BAR (OR = 1.151; 95% CI = 1.056 − 1.256; *p* = 0.001) and UHR (OR = 2.257; 95% CI = 1.507 − 3.382; *p* < 0.001) were the most important predictors for the diagnosis of OSAS, while CAR (OR = 0.999; 95% CI = 0.951 − 1.049; *p* = 0.953), RAR (OR = 1.316; 95% CI = 0.884–1.960; *p* = 0.176), and NAR (OR = 0.846; 95% CI = 0.562–1.273; *p* = 0.421) did not show significant associations (Table 4). In contrast, RAR (OR = 1.844; 95% CI = 1.224 − 2.778; *p* = 0.003) and UHR (OR = 2.203; 95% CI = 1.496 − 3.243; *p* < 0.001) were identified as the most important indices in predicting severe (OSAS) (Table 4).

## 4. Discussion

In this research, we evaluated the use of several laboratory biomarkers and hematological indices as potential obstructive sleep apnea syndrome (OSAS) screening tools. In our study, the BUN-to-albumin ratio (BAR), RDW-to-albumin ratio (RAR), and uric acid-to-HDL ratio (UHR) were found to be important predictive biomarkers for OSAS diagnosis, severity, and screening. Hematological biomarkers and various indices can be used as inexpensive and easily accessible tools in OSAS screening and diagnosis to identify patients at risk who should be referred for polysomnography (PSG).

OSAS is a highly prevalent, underdiagnosed disease. Conventional-type diagnostic methods such as polysomnography persist as the best guide, but are often time-demanding, high-cost, and not accessible to numerous individuals [26]. The AASM has reported that blood markers may be valuable in assessing patients at potential risk for OSAS [6]. Initiating OSAS biomarker analysis in potentially at-risk groups may significantly improve earlier and more accurate referral to polysomnography in groups with a higher likelihood of having OSAS or severe OSAS [27]. Considering that approximately 30–50% of patients diagnosed with OSAS are not obese and the vast majority remain undiagnosed, these hematologic parameters, as a successful screening test, allow the early diagnosis of OSAS groups other than the classically obese and male.

Previous studies have indicated that inflammatory parameters are markedly elevated in individuals with OSAS, and many hematologic parameters and indices have yielded different results in various studies. Recent studies suggest that several inflammatory markers are also associated with disease or disease severity [8,28].

It is known that OSAS is correlated with many diseases, such as cardiovascular diseases, arterial hypertension, dyslipidemia, obesity, and diabetes, as well as increasing morbidity and mortality. The underlying mechanisms are thought to be sympathetic nervous system hyperactivity due to intermittent hypoxia, an oxidative stress state, dysfunction of the vascular endothelium, and endocrine–metabolic dysregulation [29,30].

Besides inflammation of the respiratory tract, high levels of markers of systemic inflammation have been found in OSAS patients [31]. The neutrophil-to-lymphocyte ratio (NLR) is widely employed in the clinical assessment of inflammation-related diseases. The platelet-to-lymphocyte ratio (PLR), NLR, and CRP-to-albumin ratio (CAR) are used as biomarkers associated with many inflammatory diseases and their severity [32]. The NLR (which is associated with inflammation and is a prognosis-related marker in many studies), CRP (a protein that increases in the acute-phase response), and CAR (which is defined as a negative acute-phase protein and is inversely correlated with the degree of inflammatory response) are currently being investigated with great interest [16]. The NLR and CAR have been related to the outcomes of many infectious and non-infectious diseases, such as sepsis and cancer. The ratio of CRP to prealbumin is not associated with the degree of severity of OSAS and has a weak relationship with the AHI [33]. In our research, the NLR and PLR were not associated with OSAS and its severity. The CAR was markedly elevated in individuals with OSAS, but not in severe OSAS; it was not a predictor for OSAS and its severity in our study. Albumin, an important acute-phase reactant, is used as an important biomarker in the diagnosis of various diseases such as pancreatitis and acute myocardial infarction [6,20,21]. BUN has also been reported to be strongly related to the disease state severity, and recent studies have emphasized that the BUN-to-albumin ratio is a sensitive marker of mortality and morbidity [34]. However, no study on the BUN-to-albumin ratio (BAR) in OSAS was found in the literature. In our research, the BAR was markedly increased in individuals with OSAS and was found to be an important determinant of the presence of OSAS. The importance of the neutrophil-to-albumin ratio (NAR) as a biomarker for predicting prognosis in the presence of severe inflammation has been frequently recognized in recent years [35]. However, in our study, the NAR was significantly increased in patients with severe OSAS but was not determinant for the diagnosis and degree of OSAS.

Erythrocyte distribution width and serum albumin levels have emerged as important parameters reflecting systemic inflammation and general physiological status. Decreased serum albumin levels and an increased RDW have been related to inflammation, oxidative stress, and malnutrition [36]. The RDW-to-albumin ratio (RAR) has been proposed as a new prognostic biomarker. The RAR has demonstrated prognostic value in various cardiovascular disorders and stroke and has been reported to be associated with a significantly increased morbidity rate and greater mortality [37]. In our study, the RDW-to-albumin ratio (RAR) was elevated in OSAS and severe OSAS patients and was found to be an important marker for severe OSAS.

Uric acid is an important oxidative stress marker. The cycle of decreasing oxygen saturation and reoxygenation during sleep increases oxidative stress. Uric acid is a stronger and independently predictive biomarker of increased morbidity and mortality in diseases such as cardiovascular disorders, chronic renal failure, and metabolic syndrome [37]. The uric acid–HDL ratio (UHR) has been reported as a potential new marker predicting oxidative stress and metabolic imbalances. Academic studies have shown that the UHR is well linked to various diseases, such as diabetes mellitus, kidney disease, non-alcoholic fatty liver disorder, and metabolic imbalance. However, its application in OSAS studies is still evolving [38]. This supports that the UHR is a suitable diagnostic biomarker for early diagnosis and risk assessment in OSAS patients. In our study, the UHR was observed to be statistically increased in OSAS and non-OSAS, OSAS severity, and severe OSAS and non-OSAS groups, and it was reported to be an important predictor of the presence of both OSAS and severe OSAS.

Cells such as monocytes and macrophages also play an important role in the inflammatory process. They contribute to the secretion of pro-inflammatory cytokines in inflamed tissues. HDL is a molecule with an anti-inflammatory effect. The monocyte–HDL cholesterol ratio (MHR) is a recently used biomarker of inflammation. A recently conducted study showed a relationship between an elevated MHR and OSAS severity [9]. LDL cholesterol, unlike HDL cholesterol, is a well-established biochemical indicator with oxidative activity [39]. Nevertheless, very few academic studies in the literature have examined the relationship between monocytes, LDL cholesterol, and OSAS severity. In our study, the MLR and MHR were not statistically significant when comparing OSAS and severity.

Hemoglobin and RDW have also been associated with oxidative stress, inflammatory response, and the prognosis of some malignancies. However, although some researchers have suggested that Hb can be employed to evaluate OSAS severity and predict medical complications, other studies have found no such rise in Hb in individuals with OSAS [22]. Although the HRR has been proven to be predictive in conditions such as malignancies and rheumatoid arthritis, its role in OSAS has not been investigated much [40]. Earlier studies suggest that RDW is increased in individuals with OSAS and positively related to the AHI, suggesting that it may indicate disease severity. This supports the HRR as a non-invasive, reliable marker for OSAS severity [24]. In this study, the RDW was markedly higher in the severe OSAS groups, and Hb was significantly higher in the OSAS groups. In contrast, the HRR was not significant in group comparisons for the presence and severity of OSAS.

This is the first study to comprehensively evaluate the relationship between the presence and severity of OSAS and new hematological indices derived from numerous hematological biomarkers and their ratios. As far as we know, it is also the first study to examine the NAR, RAR, and BAR in OSAS. The potential clinical impact of these markers is very promising, but further studies are needed to confirm their usefulness and establish standard cut-off values for clinical application. Future studies should focus on large-scale, multi-center, prospective investigations in different population groups. The strengths of our study include the application of PSG to all participants and the provision of several new potential tools for determining the severity of OSAS and improving OSAS screening and accuracy.

The limitations of our study include its single-center nature, its retrospective design, and the failure to exclude confounding factors such as obesity, comorbidities, and anthropometric measurements. Blood biomarkers may be influenced by other factors not analyzed in this assessment, such as obesity, comorbidities, and medication regimens. The lack of adjustment with confounders in the determination of laboratory indices affecting OSAS can be considered as a limitation. To further validate our findings, prospective, multi-center studies incorporating additional clinical data, such as anthropometric measurements, snoring, sleepiness scales, and comorbidities, as well as positive airway pressure (PAP) therapy and follow-up, are required.

## 5. Conclusions

Blood biomarkers and indices associated with OSAS provide a novel approach to OSAS screening. Increased BAR, RAR, and UHR levels should first raise a high probability of OSAS and suspicion of severe OSAS and should be considered for use as an initial OSAS screening tool that may eventually lead to a sleep study referral. Biomarkers can help identify and categorize patients at higher risk for OSAS diagnosis and treatment.

## Figures and Tables

**Table 1:** Comparison of demographic characteristics and laboratory findings of patients with simple snoring (control) and OSAS.

Characteristics	Simple Snoring (Control) (n: 182) Median (IQR)	OSAS (n: 431) Median (IQR)	*p*-Value
Age (mean ± SD) *	49.02 ± 12.02	55.22 ± 11.13	<0.001
Gender (female/male), n (%) **	89 (49.2%)/93 (50.8%)	158 (36.7%)/273 (63.3%)	0.003
WBCs (K/uL)	7.73 (3.00)	7.66 (2.84)	0.065
Neutrophils (K/UL)	4.11 (1.33)	4.11 (1.33)	0.988
Lymphocytes (K/UL)	2.52 (1.23)	2.48 (1.07)	0.829
Monocytes (K/UL)	0.55 (0.25)	0.56 (0.25)	0.996
Basophils (K/UL)	0.30 (0.02)	0.03 (0.03)	0.750
Eosinophils (K/UL)	0.18 (0.15)	0.17 (0.14)	0.793
RBCs (M/uL)	4.96 (0.61)	5.07 (0.65)	0.001
RDW (%)	13.50 (1.45)	13.70 (1.30)	0.060
Hb (gr/dL)	14.16 (2.40)	14.50 (2.20)	0.046
Htc (%)	41.70 (5.35)	42.90 (5.20)	0.001
MPV (fL)	10.40 (1.45)	10.40 (1.40)	0.922
MCV (fL)	85.50 (5.95)	85.05 (6.20)	0.788
PLTs (10^3/µL)	254.00 (82.50)	240.00 (70.00)	0.076
PDW (GSD)	16.70 (30.95)	14.20 (28.10)	0.179
CRP (mg/l)	3.45 (3.18)	4.00 (5.64)	0.004
BUN (mg/dL)	29.00 (10.00)	32.00 (11.00)	<0.001
Creatinine (mg/dL)	0.87 (0.24)	0.90 (0.23)	<0.001
Albumin (g/dL)	4.20 (0.46)	4.13 (0.43)	0.060
Uric acid (mg/dL)	5.00 (1.40)	5.60 (1.67)	<0.001
HDLs (mg/dL)	42.00 (15.00)	40.00 (11.00)	0.005
LDLs (mg/dL)	111.00 (43.00)	114.50 (47,00)	0.404
NLR	1.63 (0.94)	1.62 (0.89)	0.712
PLR	95.31 (60.10)	96.77 (48.73)	0.444
CAR	0.83 (0.85)	0.98 (1.40)	0.001
RAR	3.23 (0.51)	3.30 (0.52)	0.034
BAR	6.82 (2.85)	7.62 (2.83)	<0.001
NAR	0.99 (0.47)	0.97 (0.48)	0.789
MHR	0.13 (0.01)	0.01 (0.01)	0.180
MLR	0.005 (0.00)	0.005 (0.00)	0.611
UHR	0.12 (0.06)	0.14 (0.06)	<0.001
HRR	2.90 (0.25)	2.87 (0.25)	0.072

WBCs, white blood cells; RDW, red blood cell distribution width; Hb, hemoglobin; Hct, hematocrit; PDW, platelet distribution width; MCV, mean corpuscular volume; MPV, mean platelet volume; PLTs, platelets; CRP, C-reactive protein; BUN, blood urea nitrogen; HDLs, high-density lipoproteins; LDLs, low-density lipoproteins; NLR, neutrophil-to-lymphocyte ratio; PLR, platelet-to-lymphocyte ratio; RAR, RDW-to-albumin ratio; CAR, CRP-to-albumin ratio; BAR, BUN-to-albumin ratio; NAR, neutrophil-to-albumin ratio; MHR, monocyte-to-HDL ratio; MLR, monocyte-to-LDL ratio; UHR, uric acid-to-HDL ratio; HRR, hemoglobin-to-RBC ratio. Data are expressed as medians and IQRs (interquartile ranges), compared using the Mann–Whitney U test. *p*-value < 0.05 was considered statistically significant. * Independent *t*-test (mean ± SD); ** n (%).

**Table 2:** Comparison of OSAS severity groups with laboratory parameters and indices.

**Characteristics**	**Simple Snoring (n: 182)** **Median (IQR)**	**Mild OSAS** **(n: 176)** **Median (IQR)**	**Moderate OSAS** **(n: 116)** **Median (IQR)**	**Severe OSAS** **(n: 139)** **Median (IQR)**	***p*-Value**
Age (mean ± SD) *	49.02 ± 12.02	54.57 ± 11.02	54.49 ± 11.24	56.00 ± 11.11	<0.001
WBCs (K/uL)	7.73 (3.00)	7.27 (2.67)	7.41 (2.66)	8.39 (3.15)	0.043
Neutrophils (K/UL)	4.11 (1.99)	3.89 (1.80)	4.10 (1.88)	4.27 (2.00)	0.143
Lymphocytes (K/UL)	2.52 (1.23)	2.43 (1.17)	2.52 (0.91)	2.53 (1.17)	0.779
Monocytes (K/UL)	0.55 (0.25)	0.54 (0.23)	0.55 (0.25)	0.57 (0.28)	0.527
Basophils (K/UL)	0.03 (0.02)	0.03 (0.02)	0.03 (0.02)	0.04 (0.03)	0.086
Eosinophils (K/UL)	0.18 (0.15)	0.17 (0.16)	0.15 (0.14)	0.18 (0.14)	0.739
RBCs (M/uL)	4.96 (0.61)	5.00 (0.62)	5.06 (0.66)	5.12 (0.71)	<0.001
RDW (%)	13.50 (1.45)	13.60 (1.20)	13.50 (1.22)	13.90 (1.43)	<0.001
Hb (gr/dL)	14.10 (2.40)	14.40 (2.38)	14.45 (1.90)	14.55 (2.22)	0.168
Htc (%)	41.70 (5.35)	42.70 (5.68)	42.95 (4.02)	43.65 (5.08)	0.001
MPV (fL)	10.40 (1.45)	10.30 (1.40)	10.30 (1.50)	10.50 (1.50)	0.713
MCV (fL)	85.50 (5.95)	85.15 (5.95)	84.80 (5.85)	85.30 (6.42)	0.803
PLTs (10^3/µL)	254.00 (82.50)	238.00 (72.00)	236.00 (84.25)	253.00 (82.25)	0.057
PDW (GSD)	16.70 (30.95)	14.20 (26.80)	15.00 (30.17)	13.95 (30.20)	0.239
CRP (mg/l)	3.55 (3.18)	3.77 (4.08)	4.82 (6.24)	3.98 (5.85)	0.015
BUN (mg/dL)	29.00 (10.00)	31.00 (12.00)	32.00 (9.00)	32.50 (10.00)	<0.001
Creatinine (mg/dL)	0.87 (0.24)	0.91 (0.23)	0.91 (0.21)	0.88 (0.27)	<0.001
Albumin (g/dL)	4.20 (0.46)	4.20 (0.40)	4.10 (0.50)	4.10 (0.40)	0.035
Uric acid (mg/dL)	5.00 (1.40)	5.55 (1.60)	5.60 (1.80)	5.90 (1.90)	<0.001
HDLs (mg/dL)	42.00 (15.00)	41.00 (11.00)	39.00 (10.00)	40.00 (10.00)	0.001
LDLs (mg/dL)	111.00 (43.00)	116.00 (47.00)	116.50 (46.25)	111.00 (46.50)	0.353
NLR	1.63 (0.94)	1.57 (1.09)	1.63 (0.85)	1.63 (0.84)	0.846
PLR	95.31 (60.10)	96.27 (57.90)	92.20 (47.87)	98.74 (41.49)	0.705
CAR	0.83 (0.85)	0.93 (1.03)	1.14 (1.62)	0.99 (1.42)	0.801
RAR	3.23 (0.51)	3.25 (0.50)	3.31 (0.57)	3.39 (0.67)	0.001
BAR	6.82 (2.85)	7.52 (2.77)	7.76 (2.73)	7.64 (3.40)	<0.001
NAR	0.99 (0.47)	0.93 (0.46)	0.96 (0.45)	1.02 (0.47)	0.059
MHR	0.01 (0.01)	0.01 (0.01)	0.01 (0.01)	0.01 (0.01)	0.065
MLR	0.005 (0.00)	0.004 (0.00)	0.004 (0.00)	0.005 (0.00)	0.748
UHR	0.122 (0.06)	0.138 (0.06)	0.137 (0.06)	0.151 (0.07)	<0.001
HRR	2.90 (0.25)	2.88 (0.22)	2.88 (0.26)	2.85 (0.24)	0.065

WBCs, white blood cells; RDW, red blood cell distribution width; Hb, hemoglobin; Hct, hematocrit; PDW, platelet distribution width; MCV, mean corpuscular volume; MPV, mean platelet volume; PLTs, platelets; CRP, C-reactive protein; BUN, blood urea nitrogen; ALT, alanine aminotransferase; ALP, alkaline phosphatase; HDLs, high-density lipoproteins; LDLs, low-density lipoproteins; NLR, neutrophil-to-lymphocyte ratio; PLR, platelet-to-lymphocyte ratio; RAR, RDW-to-albumin ratio; CAR, CRP-to-albumin ratio; BAR, BUN-to-albumin ratio; NAR, neutrophil-to-albumin ratio; MHR, monocyte-to-HDL ratio; MLR, monocyte-to-LDL ratio; UHR, uric acid-to-HDL ratio; HRR, hemoglobin-to-RBC ratio. Data are expressed as medians and IQRs (interquartile ranges), compared using the Mann–Whitney U test. *p*-value < 0.05 was considered statistically significant. * Independent *t*-test (mean ± SD).

**Table 3:** Comparison of laboratory characteristics between severe and non-severe OSAS.

**Characteristics**	**Non-Severe OSAS (n: 292)** **Median (IQR)**	**Severe OSAS (n: 139)** **Median (IQR)**	***p*-Value**
Age (mean ± SD) *	54.54± 11.08	56.00 ± 11.11	0.102
WBCs (K/uL)	7.34 (2.63)	8.39 (3.15)	0.005
Neutrophils (K/UL)	3.99 (1.88)	4.27 (0.03)	0.029
Lymphocytes (K/UL)	2.45 (1.03)	2.53 (1.17)	0.580
Monocytes (K/UL)	0.55 (0.23)	0.57 (0.28)	0.322
Basophils (K/UL)	0.03 (0.02)	0.04 (0.03)	0.020
Eosinophils (K/UL)	0.17 (0.15)	0.18 (0.14)	0.427
RBCs (M/uL)	5.03 (0.67)	5.12 (0.71)	0.047
RDW (%)	13.60 (1.23)	13.90 (1.43)	<0.001
Hb (gr/dL)	14.40 (2.20)	14.55 (2.22)	0.522
Htc (%)	42.75 (5.05)	43.65 (5.08)	0.021
MPV (fL)	10.30 (1.40)	10.50 (1.50)	0.368
MCV (fL)	85.00 (6.10)	85.30 (6.42)	0.788
PLTs (10^3/µL)	237.00 (74.25)	253.00 (82.25)	0.046
PDW (GSD)	14.40 (27.75)	13.95 (30.20)	0.939
CRP (mg/l)	4.01 (5.35)	3.98 (5.85)	0.916
BUN (mg/dL)	32.00 (10.25)	32.50 (10.00)	0.352
Creatinine (mg/dL)	0.91 (0.22)	0.88 (0.27)	0.803
Albumin (g/dL)	4.20 (0.40)	4.10 (0.40)	0.087
Uric acid (mg/dL)	5.60 (1.63)	5.90 (1.90)	0.009
HDLs (mg/dL)	40.00 (11.25)	40.00 (10.00)	0.027
LDLs (mg/dL)	116.00 (46.25)	111.00 (46.50)	0.309
NLR	1.62 (0.97)	1.63 (0.84)	0.407
PLR	95.06 (53.57)	98.74 (41.49)	0.772
CAR	0.97 (1.29)	0.99 (1.42)	0.596
RAR	3.27 (0.54)	3.39 (0.67)	0.001
BAR	7.61 (2.75)	7.64 (3.40)	0.230
NAR	0.95 (0.48)	1.02 (0.47)	0.012
MHR	0.01 (0.01)	0.01 (0.01)	0.079
MLR	0.004 (0.00)	0.005 (0.00)	0.336
UHR	0.13 (0.06)	0.15 (0.07)	0.002
HRR	2.88 (0.24)	2.85 (0.24)	0.052

WBCs, white blood cells; RDW, red blood cell distribution width; Hb, hemoglobin; Hct, hematocrit; PDW, platelet distribution width; MCV, mean corpuscular volume; MPV, mean platelet volume; PLTs, platelets; CRP, C-reactive protein; BUN, blood urea nitrogen; HDLs, high-density lipoproteins; LDLs, low-density lipoproteins; NLR, neutrophil-to-lymphocyte ratio; PLR, platelet-to-lymphocyte ratio; RAR, RDW-to-albumin ratio; CAR, CRP-to-albumin ratio; BAR, BUN-to-albumin ratio; NAR, neutrophil-to-albumin ratio; MHR, monocyte-to-HDL ratio; MLR, monocyte-to-LDL ratio; UHR, uric acid-to-HDL ratio; HRR, hemoglobin-to-RBC ratio. Data are expressed as medians and IQRs (interquartile ranges), compared using the Mann–Whitney U test. *p*-value < 0.05 was considered statistically significant. * Independent *t*-test (mean ± SD).

**Table 4:** Multivariate logistic regression analyses for laboratory parameters predicting OSAS diagnosis and severe OSAS.

**OSAS–Control (SS)**	**B**	***p*-Value**	**OR**	**95% CI**	
				**Lower**	**Upper**
CAR	−0.001	0.953	0.999	0.951	1.049
RAR	0.275	0.176	1.316	0.884	1.960
BAR	0.141	0.001	1.151	1.056	1.256
NAR	−0.168	0.421	0.846	0.562	1.273
UHR	0.814	<0.001	2.257	1.507	3.382
**Severe–Non-severe OSAS**	**B**	***p*-value**	**OR**	**95% CI**	
				**Lower**	**Upper**
CAR	−0.041	0.205	0.960	0.902	1.023
RAR	0.612	0.003	1.844	1.224	2.778
BAR	−0.008	0.839	0.992	0.917	1.073
NAR	0.178	0.437	1.195	0.762	1.874
UHR	0.790	<0.001	2.203	1.496	3.243

SS, simple snoring; CAR, CRP-to-albumin ratio; RAR, RDW-to-albumin ratio; BAR, BUN-to-albumin ratio; NAR, neutrophil-to-albumin ratio; UHR, uric acid-to-HDL ratio; B, standardized regression coefficient; OR, odds ratio; CI, confidence interval.

## Data Availability

The data supporting the findings of this study are available upon reasonable request from the corresponding author. The data are not publicly available due to privacy or ethical restrictions.
